# The German version of the Pregnancy Physical Activity Questionnaire: a translation, cross-cultural adaptation, reliability and validity assessment

**DOI:** 10.1186/s12884-024-06804-5

**Published:** 2024-09-17

**Authors:** Mark Spiller, Nina Ferrari, Christine Joisten

**Affiliations:** 1 Department for Physical Activity in Public Health, Institute of Movement and Neurosciences, German Sport University Cologne, Am Sportpark Müngersdorf 6, Cologne, 50933 Germany; 2 Cologne Center for Prevention in Childhood, Youth/Heart Center Cologne, University Hospital of Cologne, Kerpener Str. 62, Cologne, 50937 Germany

**Keywords:** Activity Assessment, Energy Consumption in metabolic equivalents, Cultural Adaptation, Translation, Reproducibility of results, German population

## Abstract

**Background:**

Validated and internationally standardised measurement instruments are a prerequisite for ensuring that physical activity during pregnancy is comparable and for deriving physical activity recommendations. In Germany, there has been no adapted version of the internationally used Pregnancy Physical Activity Questionnaire (PPAQ) until now. This study’s aim centred around translating the original English version into German (PPAQ-G) and determining its reliability as well as validity in a German population.

**Methods:**

The PPAQ was translated into German using the forward-backwards technique. Its reliability and validity were tested. Thirty-four correctly completed questionnaires were analysed. The test–retest reliability was presented using the intraclass correlation coefficient (ICC) and Spearman correlation coefficient. Validity was tested by using accelerometer (*n* = 23) and determined by Spearman correlation coefficient.

**Results:**

In the transcultural adjustment, two questions were amended to describe intensity more precisely, and two other questions were adapted to reflect the units of measurement used in Germany. The ICC indicated a reliability of *r* = 0.79 for total activity (without sitting), and the intensity subcategories ranged from *r* = 0.70 (moderate-intensity activities) to *r* = 0.90 (sitting). Although, validity assessment showed no significant correlation for sedentary, moderate or vigorous intensity, there were significant correlations for total activity (light and above; *r* = 0.49; *p* < 0.05) and for light activity (*r* = 0.65; *p* < 0.01).

**Conclusions:**

The PPAQ-G showed good reliability for use on pregnant German women and a moderately accurate measurement of physical activity. It can be used nationally for epidemiological studies, and it also enables international comparisons of physical activity during pregnancy.

**Trial registration:**

DRKS00023426; Registration date 20 May 2021.

**Supplementary Information:**

The online version contains supplementary material available at 10.1186/s12884-024-06804-5.

## Background

 Physical activity during and after pregnancy has several positive effects on maternal health [1, 2] During pregnancy regular physical activity can help to maintain fitness [3, 4] and protects against excessive weight gain [5, 6]. It also reduces the risk of developing gestational diabetes [7–11]. Moreover, physical activity also reduces the number of caesarean deliveries and promotes better postnatal rehabilitation [4].

Daily physical activity of at least 20–30 min at a moderate intensity (analogically 150 min per week) is recommended for pregnant women both internationally [12–16] and in Germany [17]. However, only a few pregnant women meet the current activity recommendations, with the percentage varying depending on the method which was used to gain the data: For example, telephone interviews were used to determine that 11% of pregnant women met activity recommendations [18], and questionnaires were employed to ascertain that 16–31% of pregnant women met these recommendations [19–21]. By using objectively measured physical activity (accelerometer) Evenson & Wen [22] revealed, that most of the studied pregnant women spent more than 50% of the monitored day in sedentary behaviors and did not meet recommendations for physical activity.

Recommendations should be based on validated measurement instruments, but there is no existing gold standard. However, the internationally renowned and validated Pregnancy Physical Activity Questionnaire (PPAQ) developed by Chasan-Taber et al. [23] is often used as it is used to record the duration, frequency, and intensity of physical activity in which a woman engages as well as her current trimester. The activities are divided into five categories: household and caregiving (12 activities); occupation (five activities); sports and exercise (nine activities); transportation (three activities); and inactivity (three activities). They are created based on Ainsworth et al.’s [24] compendium, which calculates average daily energy expenditure (metabolic equivalents [METs] × hours/day) by multiplying self-reported time spent on each activity by activity intensity (in METs) to achieve the following classifications: sedentary (< 1.5 METs); light intensity (1.5 ≤ 3.0 METs); moderate intensity (3.0–6.0 METs); and vigorous intensity (< 6.0 METs). The time spent sleeping is not included in the calculation.

The PPAQ [23] has been translated into twelve languages: Vietnamese [25]; Japanese [26]; Canadian French [27]; Turkish [28], Brazilian Portuguese [29]; Portuguese [30]; Chinese [31], Polish [32, 33]; Serbian [34]; Spanish [35, 36]; Arabic [37], and Danish [38, 39]. Thus, a German version of the PPAQ does not yet exist.

There are nine of the translated 12 versions that tested the reliability and used intraclass correlation coefficient (ICC), with good results being obtained for total activity (without sitting) as the reliability ranged from *r* = 0.75 [32] to *r* = 0.95 [30]. Nine of 12 studies that translated the questionnaire also assessed the validity by using accelerometers [23, 26, 27, 30, 31], pedometers [25, 28], the International Physical Activity Questionnaire long version (IPAQ) [28, 34], and a 24-hour multi-sensor monitor [36]. Two studies used eye validity for testing the validity of the transcultural adaptation. In one study, the questionnaires’ content was reviewed by experts under clinical supervision [25], and another study asked 10 pregnant women about their understanding of the questionnaire post-completion as part of a pre-study [38].

The aim of the present study was to translate the PPAQ into German (PPAQ-G), to adapt it to national conditions and, based on this, to test its reliability and validity in order to develop an assessment tool for measuring physical activity during pregnancy in Germany.

## Methods

This study was conducted from February 2020 – November 2022 and comprised three parts: Firstly, the PPAQ was translated and transculturally adapted from the original English version into German through a forward-backwards translation, with consent for the translation being obtained in advance from the author of the original [23] ; and, secondly, 40 pregnant women completed the questionnaire twice over seven days (i.e. the test–retest method). Thirdly, these women were asked to wear an accelerometer (ActiGraph wGT3X-BT; (ActiGraph, LLC, Pensacola, US)) for seven days to compare data from the PPAQ-G with objectively measured values. The study was approved by the Ethics Committee of the German Sport University Cologne (ethics reference number: 140/2020; date of approval: 12.10.2020), and all study participants provided written informed consent confirming their voluntary participation. The final version of the German translation of the PPAQ can be found in the additional file (Additional file 1).

### The translation and transcultural adaptation of the PPAQ

The translation and transcultural adaptation of the PPAQ was guided by Guillemin et al.’s [40] quality guidelines. Forward-backwards translation is the minimum requirement for cross-cultural adaptation of scientific texts [41].

#### Phase 1: translation

The PPAQ was translated into German by a sports scientist (PhD), who has many years of experience with physical activity questionnaires.

#### Phase 2: backwards translation

After the PPAQ was translated into German, it was back-translated into English by a native English speaker. The native speaker was not informed about the original version of the PPAQ nor the study’s purpose.

#### Phase 3: committee review

An interdisciplinary team comprising a medical doctor, two sports scientists, and two research assistants jointly reviewed the translation and back-translation. They then compared the translated version to the original version and discussed the results with the native English speaker. The comprehensibility and usability of the translation were reviewed point by point.

Four questions were changed in the transcultural adaptation. The first and second changes comprised adding intensities for the questions regarding gardening: Question 18 was originally ‘Mowing the lawn while on a riding mower’, which was changed to ‘Lightly working in the garden, such as mowing the lawn on a riding mower’ (question 15 in the PPAQ-G); and the original ‘Mowing the lawn using a walking mower, raking, gardening’ (question 19 in the PPAQ) became ‘Working moderately in the garden – mowing or raking the lawn’ (question 16 in the PPAQ-G). The third and fourth amendments related to units of measurement as ‘gallon’ was changed to ‘kilogram’ in the PPAQ-G due to this being the unit of measurement commonly used for weight in Germany: Question 33 in the original questionnaire, ‘Standing or walking slowly at work while carrying things (heavier than a one-gallon milk jug)’, was changed to ‘To stand or walk slowly while carrying things heavier than 4 kg’ in question 30 of the PPAQ-G; and ‘Walking quickly at work while carrying things (heavier than a one-gallon milk jug)’, which was question 35 in the PPAQ, became ‘Walking quickly while carrying things heavier than 4 kg’ in question 32 of the PPAQ-G.

#### Phase 4: final version preparation

Finally, the entire questionnaire and all the changes were reviewed again, after which the final version of the PPAQ-G was produced.

### The reliability testing of the PPAQ-G

#### Study population

Pregnant women over 18 were included in the study, while pregnant women with possible contraindications to physical activity [14], with insufficient knowledge of German, and without a signed informed consent form were excluded. A total of 40 pregnant women were recruited for the study from a prenatal medicine practice or gynaecologist practices. Six women were excluded due to their not completing the questionnaires. Overall, data from 34 women aged 26–41 (33.6 ± 3.8) who were in their first (*n* = 2), second (*n* = 21), and third (*n* = 11) trimesters of pregnancy were analysed.

#### Additional data

Alongside the PPAQ-G, anthropometric data that included health-related parameters were collected via questionnaire. These data comprised the participants’ date of birth, height, weight (current and pre-pregnancy) and week of pregnancy. Their BMI was calculated as the ratio between their weight (before pregnancy) in kilograms and their height squared in metres, with the participants being categorised according to the World Health Organisation’s guidelines: a BMI of < 18.5 kg/m² is considered underweight; a BMI of 18.5–24.9 kg/m² denotes a normal weight; and a BMI of ≥ 25 kg/m² is categorised as overweight or obese [42, 43].

#### The reliability test

The test–retest method was used to assess reliability: All the participants received two PPAQ-G questionnaires and were asked to complete them seven days apart. There was no time limit for the completion of the questionnaires.

### The validity testing of the PPAQ-G

#### Study population

Of the total of 40 participants, eleven women were excluded due to non-availability of accelerometer data. A further six data sets were excluded due to the accelerometer not being worn long enough per day. Only those participants were included in the validity-study who wore the accelerometer at least 480 min per day [30, 44]. We considered women with at least 3 days of measurement to have valid data for inclusion [30, 45]. The final data were therefore available from 23 test subjects aged 27–41 (34.4 ± 3.6) years during their first (*n* = 1), second (*n* = 16) or third (*n* = 6) trimester of pregnancy.

#### Actigraphy

The ActiGraph wGT3X-BT (ActiGraph, LLC, Pensacola, US) was worn around the woman’s waist for seven consecutive days between completing the questionnaires. The data were collected in 1-minute activity counts. The data obtained by the accelerometer were downloaded and analyzed by a program provided by the manufacturer, the Actilife software.

Cut-points from Hendelman et al. [46] were used to classify the activity level detected by the ActiGraph. Cut-points are as followed: <100 counts/min (sedentary activity); 100–1,951 counts/min (light-intensity activity); 1,952–5,724 counts/min (moderate-intensity activity); >5,724 counts/min (vigorous-intensity activity).

### Statistical analysis

The statistical analysis was conducted using *SPSS Statistics* 29 (IBM, Corp., Armonk, NY). The descriptive statistics were displayed as mean ± standard deviation. The median was calculated for the continuous variables, and the number of subjects and percentages were calculated for the categorical variables. By using the Kolmogorov-Smirnov test the normal distribution was tested. Normally distributed parameters were analyzed with parametric tests. A two-sample t-test for dependent variables was applied to assess whether activity values (in MET/h.week-1) from the first and second PPAQ-G had the same or different means. If parameters were not normally distributed the Wilcoxen-Test was used.

The study’s reliability was assessed by measuring the one-week test–retest reliability. Due to not normally distributed data, data was transformed using the Box-Cox transformation. The two-way mixed effects ICC model and Spearman correlation coefficient were used to assess the reliability in the respective life domains and intensity categories. All confidence intervals were estimated at a level of 95%, and a p-value of < 0.05 indicated statistical significance.

The values of the two correlation coefficients can range from 0 to 1 (or -1 for the Spearman correlation coefficient) in which 0 represents no inter-rater reliability and 1 and − 1 both represent perfect inter-rater reliability [47]. The ICC scores were derived using Koo’s and Li’s [47] classification: *r* < 0.50 was poor; 0.50–0.75 was average; 0.75–0.90 was good; and *r* > 0.90 was very good.

The median values in the unit MET/h. week-1 for the first and second PPAQ-G were calculated for the total activity (excluding sitting), the individual intensity categories, and the three life domains (i.e. household and caregiving, occupation, sports and exercise). In addition, the 25th and 75th percentiles are presented to show the wide range of our values. The intensity categories were based on the original PPAQ guidelines set by Chasan-Taber et al. [23], who recommended classifying participants into the different MET categories.

Spearman correlation coefficients were calculated between the PPAQ-G and the accelerometer values to evaluate the validity of the PPAQ-G.

## Results

### The study population

Table 1 shows the participants’ anthropometric characteristics. The participants’ mean age was 33.6 ± 3.8 years, and their mean pre-gestational BMI was 23.3 ± 4.4 kg/m^2^. Those who were underweight (BMI < 18.5 kg/m^2^) accounted for 8.8% of the sample (*n* = 3), 70.6% (*n* = 24) were of a normal weight (BMI of 18.5–24.9 kg/m^2^), and 20.6% of the pregnant women (*n* = 7) were overweight or had obesity (BMI ≥ 25 kg/m^2^).

**Table 1:** The participants’ anthropometric data (*n* = 34)

	Mean ± SD	Min.	Max.
Age (years)	33.6 ± 3.8	26.0	41.0
Height (centimeters)	168.4 ± 6,3	156.0	180.0
Weight (kilograms)	72.8 ± 15.1	48.0	112.0
Gestational week	22.1 ± 7.7	9.0	38.0
Pre-pregnancy BMI (kg/m^2^)	23.3 ± 4.4	17.2	34.4

*BMI *body mass index, *n* number, *Mean ± SD* mean ± standard deviation, *Min.* minimum, *Max.* maximum

### The median values of the PPAQ-G

The analysis of the two questionnaires revealed comparable median values for total activity (excluding sitting; 133.2 versus 132.1 MET-h·wk-1) and the different activity intensities and types (see Table 2). According to their statements in the first PPAQ-G, the women sat for an average of 8.5 ± 4.0 h per day, their average weekly share of sedentary activities was 52.7% (corresponding to a median of 60.4 h/week), and their light-intensity activities accounted for 34.4% of their weekly activity (37.1 h/week). On average, moderate- or vigorous-intensity activities constituted 12.9% (12.1 h/week) of the women’s total weekly activity.

**Table 2:** Median values (as well as 25th and 75th percentiles) MET-h·wk-1 for the first and second PPAQ-G and potential significant differences (*n* = 34)

	**1st PPAQ-G** **(MET-h·wk-1)**			**2nd PPAQ-G** **(MET-h·wk-1)**			**significance**
	**25th percentile**	**Median**	**75th percentile**	**25th percentile**	**Median**	**75th percentile**	***p*-value**
Total activity values							
Total activity (light intensity and above)	80.7	133.2	173.9	80.5	132.1	201.5	0.571*
By intensity							
Sedentary (< 1.5 METs)	42.9	78.2	103.5	47.3	81.2	114.4	0.468*
Light (1.5–<3.0 METs)	54.7	85.8	111.9	49.7	94.1	118.6	0.911^#^
Moderate (3.0–6.0 METs)	24.6	40.8	68.5	24.8	34.8	76.5	0.293^#^
Vigorous (> 6.0 METs)	0.0	0.0	0.4	0.0	0.0	1.6	0.889^#^
By type							
Household/caregiving	43.1	67.4	112.4	37.8	70.1	123.6	0.285^#^
Occupation	0.0	71.1	84.1	0.0	69.4	82.4	0.910^#^
Sports/exercise	7.9	16.8	24.9	6.1	12.0	21.3	0.454^#^

*METs *metabolic equivalents, *PPAQ-G* German version of the Pregnancy Physical Activity Questionnaire

*paired ***t***-test

^#^Wilcoxen-Test

### The reliability as determined by the ICC

The ICC was used to assess the reliability in the respective life domains, intensity categories, and total activity. The reliability was *r* = 0.79 for total physical activity (without sitting), which is considered good [47]. The reliability was slightly higher for low-intensity activities, such as sitting (*r* = 0.90), than for moderate-intensity activities (*r* = 0.70). Very high reliability (*r* = 0.93) was obtained for everyday activities. Sports and exercise (*r* = 0.76) had a good reliability. In addition, the Spearman correlation coefficient was calculated for the various categories. The reliability for total physical activity (without sitting) was *r* = 0.76 (*p* ≤ 0.001), which is considered very good [48]. However, the range between the upper and lower confidence interval was high. The reliability results are shown in Table 3.

**Table 3:** The test–retest reliability results of the PPAQ-G (*n* = 34)

	Test–retest reliability	
	ICC (95% CI)	*r* (95% CI)
Total activity (light intensity and above)	0.79 (0.57–0.90)	0.76 (0.56–0.88)**
By intensity		
Sedentary (< 1.5 METs)	0.90 (0.81–0.95)	0.77 (0.57–0.88)**
Light (1.5 - <3.0 METs)	0.81 (0.62–0.91)	0.77 (0.58–0.88)**
Moderate (3.0–6.0 METs)	0.70 (0.38–0.85)	0.63 (0.36–0.80)**
Vigorous (> 6.0 METs)^a^	-	0.82 (0.66–0.91)**
By type		
Household/caregiving	0.93 (0.86–0.97)	0.91 (0.83–0.96)**
Occupation^a^	-	0.56 (0.23–0.76)**
Sports/exercise	0.76 (0.50–0.88)	0.66 (0.41–0.82)**

ICCs were calculated on Box-Cox transformed data

*PPAQ-G* German version of the Pregnancy Physical Activity Questionnaire, *r* Spearman correlation coefficient, *CI* confidence interval, *ICC* intraclass correlation coefficient, *METs* metabolic equivalents

** *p*  ≤ 0.001

^a^data not normally distributed

**Fig. 1:**
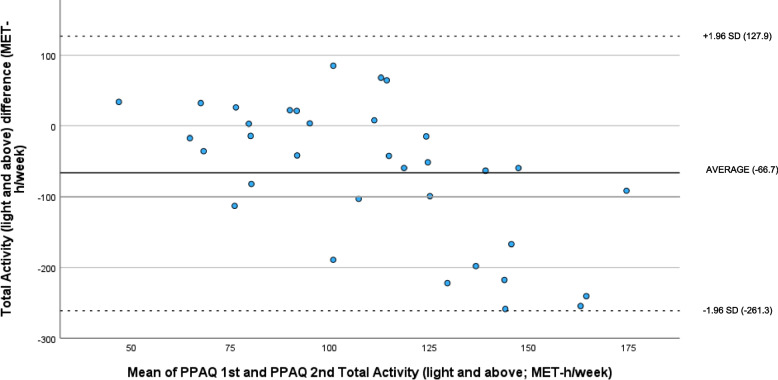
Bland-Altman plot of PPAQ 1st and PPAQ 2nd Total Activity score (light intensity and above)

By using the Bland-Altman plot (see Fig. 1) the degree of agreement between the first PPAQ assessment and the second PPAQ assessment according to total activity (light intensity and above) is illustrated. The limits of agreement were calculated as the mean difference between the analysed indices ± 1.96 for the standard deviation of the difference. The average value of the difference was − 66.7.

### Validity of the PPAQ-G

The Spearman correlation coefficient was used to assess the validity of the PPAQ-G between the PPAQ-G completed at the second visit and the average accelerometer measurement data.

There were significant correlations for total activity without sitting (light intensity and above) (*r* = 0.49; *p* < 0.05) and light activity (*r* = 0.65; *p* < 0.01) (Table 4).

**Table 4:** Spearman correlation coefficients between the pregnancy physical activity questionnaire (PPAQ) at the second visit and accelerometer data (*n* = 23)

2nd PPAQ-G measures	Actigraph Measures Actigraph cut-points (hr-week^−1^)
Activity values	
Activity (light intensity and above)	0.49*
By intensity	
Sedentary (< 1.5 METs)	0.28
Light (1.5–<3.0 METs)	0.65**
Moderate (3.0–6.0 METs)	-0.17
Vigorous (> 6.0 METs)	0.08

*PPAQ-G* German version of the Pregnancy Physical Activity Questionnaire, *hr-week*^*-1*^ hours per week, *METs* metabolic equivalents

* The correlation is significant at the 0.05 level (two-sided)

** The correlation is significant at the 0.01 level (two-sided)

## Discussion

To our knowledge, this study is the first German translation of the PPAQ and its first adaptation to the German context. It also represents the PPAQ’s first reliability and validity test for German pregnant women.

Overall, the physical activity score was very high in our sample size compared to the original work by Chasan-Taber et al. However, our physical activity level is comparable with other studies [26, 28, 32] although different racial and ethnic groups need to be considered by interpretation. For example, a Japanese study by Matsuzaki et al. [26] assessed the reliability and criterion validity of the adopted PPAQ for Japanese pregnant women. The total activity score was 19.7 METs × hours/day at the first measured timepoint, which is the equivalent of 137.9 METs × hours/week. Our measured high values of total physical activity could be due to the fact that the participants wore an activity tracker (ActiGraph) between completing the questionnaires, which may have had an increased awareness of their activity and therefore participated in more physical activity.

The ICC results showed good reliability across the different intensities: 0.79 for total activity without sitting; 0.90 for sitting; 0.81 for light-intensity activities and 0.70 for moderate-intensity activities. This is comparable to other studies that have translated the PPAQ [26, 31]. For example, Chandonnet et al. [27] conducted a study in Canada with a comparable number of subjects (49 pregnant women) and came to similar results for the different intensity categories: 0.90 for total physical activity without sitting; 0.88 for sitting; 0.86 for light-intensity activities and 0.86 for moderate-intensity activities. However, it must be taken into account that the study was conducted in pregnant women with obesity and that different cut-points were used. Comparisons with other studies are therefore difficult.

Furthermore, this study’s ICC values were lower for the moderate-intensity activity category compared to light-intensity activities or sitting. This observation is consistent with the results from other studies [26, 27, 31] and contrasts with the original study [23]. Comparing this study with the original study shows that this study’s results for total time (without sitting; 0.79 vs. 0.78), sedentary (0.90 vs. 0.79) and light-intensity activities (0.81 vs. 0.78) were better than those of the original study. However, this study’s result for moderate- (0.70 vs. 0.82) -intensity activities was worse, which may be due to the pregnant women being active less frequently in these intensity range. Moreover, no ICC for vigorous-intensity activity could be detected, due to very high skewness of the data. This is corroborated by this study’s median for vigorous-intensity activities in both questionnaires, which was zero, which is a result that aligns with other validation studies [25–28, 31, 32]. The study’s findings could also be explained by age as high ICC scores (> 0.81) for moderate- and vigorous-intensity activities were found in subjects who were, on average, 4.5 years younger [23, 25, 27, 28, 32]. Furthermore, some participants in the studies mentioned above already had older children, resulting in their engaging in more light-intensity activities throughout the day.

The ICC results for activity type (i.e. household and caregiving, occupation, sports and exercise) were heterogeneous and can be classified as follows: good (0.76 for sports and exercise); and very good (0.93 for household and caregiving) [47]. Again, the ICC for occupation could not be detected, because the skewness of the data was too high and no normally distributed could be achieved. The ICC for sports and household are comparable to the study in which the PPAQ was translated into Japanese [26] and the ICC values were lowest in the categories of occupation and sports/exercise. This observation is also consistent with other studies [26, 27, 32]. Thus, the questionnaire may not optimally represent these activity types as many of the participants were no longer working (35.4% in the first PPAQ-G of this study) and more activity takes place in general daily life.

The ICC values of this study for the intensity ranges (i.e. light and moderate, ) were similar to those in the Canadian study [27]. However, the ICC value for the sports/exercise category was significantly higher in Canada. Ota et al. [25] and Oviedo-Caro et al. [36] found an ICC of *r* ≥ 0.87, and Çırak et al. [28] found an ICC of *r* ≥ 0.92, which indicate particularly good reliabilities in all the categories discussed herein. The slightly better reliability values in Ota et al.’s [25] and Oviedo-Caro et al.’s [36] studies may be due to the participants being accompanied when answering the questionnaires, whereas both questionnaires were completed alone in this study.

This study’s test–retest reliability values ranged from 0.56 to 0.91. For overall reliability (*r* = 0.76; *p* ≤ 0.001), the results were very good [48] and comparable to previous studies, thus suggesting the successful adaptation of the PPAQ to German culture and the good reliability of the PPAQ-G. However, the occupation, sports/exercise, and moderate-intensity activities categories had significantly higher dispersions, similar to the ICC scores presented earlier. However, this dispersion has also been reported by other studies (ICC dispersion > 0.30) [26, 27, 31, 32] and does not seem to be a test–retest problem specific to the German translation. A general point of discussion is, that calculations from the PPAQ were based on values from the compendium [24]. However, these values are almost not based on data from pregnant women. In the future it would be useful to generate and provide pregnancy-specific (or maybe individualized) MET values for pregnant women.

According to the validity, we found significant correlations for total physical activity (*r* = 0.49; light and above) and light intensity activity (*r* = 0.65) which could be classified as moderate to good. Similar data and comparisons can also be seen in the Chinese validation study [31], which also used accelerometer for validity testing. In contrast to our study, the original study by Chasan-Taber et al. [23] and other studies [26, 36] showed only poor to moderate validity.

### Strengths and limitations

This study’s strengths include the high adherence of the participants as there were very few dropouts (8%) and a complete dataset. However, it also has limitations. Firstly, the sample size was small. The other 14 PPAQ validation studies included an average of 111 subjects, with a minimum of 21 and a maximum of 291. Furthermore, due to the COVID-19 pandemic, respondents’ willingness to participate was relatively low, resulting in the recruitment period for participants being extended. In addition, there is a certain limitation in the collection of data. It was possible that participants copied their information from the first questionnaire. The reliability values may have been biased due to this small sample size. Moreover, our data has a certain uncertainty in the data (e.g., wide confidence limits). According to the validity of our data it must be stated, that we used cut-points which were not validated for pregnant women. This might interfere with the validity of the data. However, even the original publication used the cut-points by Hendelman et al. [23]. Therefore, our data needs to be interpreted with caution. Secondly, another important limitation of our study is the translation and cultural adaptation method. As stated, we used the forward-backward translation with only one translator. Although our translator was specialized and had a knowledge of health terminology and the content area (as recommended by Sousa et al. [49]), this minimalistic approach could lead to cultural and linguistic nuances not being sufficiently taken into account. Thirdly, selection bias could not be excluded, especially among the participants who had a significant interest in health and physical activity. This could have influenced the activity scores (MET-h·wk-1) by increasing them. This can be seen particularly in the relatively high amount of 12.9% of the total weekly activity on moderate- or vigorous-intensity activity, which represents 12.1 h/week. In addition to selection bias, overreporting of physical activity may also play a certain role in our study and should be taken into account when interpreting the data. Fourthly, the distribution of participants between trimesters was unbalanced as 61.8% of the participants were in their second trimesters. This imbalance could be one reason why so few women in our sample were active at work and very rarely engaged in vigorous-intensity physical activity. However, this meant that this study was consistent with other PPAQ reliability studies that also showed an uneven distribution of trimesters [23, 37, 38]. Nevertheless, in the future, this aspect of achieving a more balanced distribution across the trimesters should be better taken into account. Fifthly, most of the subjects came from a single large city. Therefore, the study results may not be generalisable to all German population groups. Therefore, future studies should cover more regions in Germany and the sample size should be significantly increased.

## Conclusions

Physical activity recommendations offer pregnant women essential guidance on healthy levels of physical activity. Questionnaires are an easy-to-use instrument if they are reliable and valid. This study translated and interculturally adapted the PPAQ-G, which showed good reliability for German pregnant women with a moderately accurate measurement of physical activity. This offers the possibility for using the PPAQ-G for epidemiological studies in the future. Moreover, since this is the first questionnaire for German pregnant women, it also allows for comparisons of physical activity during pregnancy between different countries, although culture-specific and ethnic aspects must always be taken into account.

## Supplementary Information

Supplementary Material 1.

## Data Availability

The datasets used and/or analysed during the current study are available from the corresponding author on reasonable request.

## Abbreviations

BMI: body mass index
COVID-19: Coronavirus disease-2019
e.g.: for example
GDM: gestational diabetes
ICC: intraclass correlation coefficient
I.e.: that means
IPAQ: International Physical Activity Questionnaire
Kg: kilograms
CI: confidence interval
Max: maximum
METs: metabolic equivalents
Min: minimum
Mean ± SD: mean ± standard deviation
n: number
PPAQ: Pregnancy Physical Activity Questionnaire
PPAQ-G: German version of the Pregnancy Physical Activity Questionnaire
r: Spearman correlation coefficient
SD: standard deviation
Sig.: Significance

## Data availability

No datasets were generated or analysed during the current study.
