# Supplementary material for: The German version of the Pregnancy Physical Activity Questionnaire: a translation, cross-cultural adaptation, reliability and validity assessment
Source: BMC Pregnancy Childbirth. 2024 Sep 17;24:604. doi: 10.1186/s12884-024-06804-5 (PMC11409628; doi:10.1186/s12884-024-06804-5)
Supplement: Supplementary file 1 — Supplementary Material 1. [file 12884_2024_6804_MOESM1_ESM.pdf]

Pregnancy Physical Activity Questionnaire

Deutsche Übersetzung

(PPAQ-G)

Mithilfe dieses Fragebogens möchten wir Ihren aktiven bzw. inaktiven Lebensstil in diesem Trimester erfassen. Bitte beantworten Sie jede Frage ehrlich. Es gibt keine richtigen oder falschen Antworten.

Bitte Ihre Codierung eintragen: ☐ ☐

I. Fragen zu Ihrem Alltag

Wenn Sie nicht arbeiten müssen, wie viel Zeit verbringen Sie normalweise mit...

1. Essen zubereiten – inklusive Tisch decken und abwaschen

- gar keine ☐
- < 1/2 Stunde pro Tag ☐
- 1/2 bis <1 Stunde pro Tag ☐
- 1 bis <2 Stunden pro Tag ☐
- 2 bis <3 Stunden pro Tag ☐
- ≥ 3 Stunden pro Tag ☐

2. Kinder anziehen, baden und füttern, während Sie <u>sitzen</u>

- gar keine ☐
- < 1/2 Stunde pro Tag ☐
- 1/2 bis <1 Stunde pro Tag ☐
- 1 bis <2 Stunden pro Tag ☐
- 2 bis <3 Stunden pro Tag ☐
- ≥ 3 Stunden pro Tag ☐

3. Kinder anziehen, baden und füttern, während Sie <u>stehen</u>

gar keine	☐
< 1/2 Stunde pro Tag	☐
1/2 bis <1 Stunde pro Tag	☐
1 bis <2 Stunden pro Tag	☐
2 bis <3 Stunden pro Tag	☐
≥ 3 Stunden pro Tag	☐

4. Mit Kindern zu spielen, während Sie <u>sitzen oder stehen</u>

gar keine	☐
< 1/2 Stunde pro Tag	☐
1/2 bis <1 Stunde pro Tag	☐
1 bis <2 Stunden pro Tag	☐
2 bis <3 Stunden pro Tag	☐
≥ 3 Stunden pro Tag	☐

5. Mit Kindern zu spielen, während Sie <u>laufen oder rennen</u>

gar keine	☐
< 1/2 Stunde pro Tag	☐
1/2 bis <1 Stunde pro Tag	☐
1 bis <2 Stunden pro Tag	☐
2 bis <3 Stunden pro Tag	☐
≥ 3 Stunden pro Tag	☐

6. Kinder zu tragen

gar keine	☐
< 1/2 Stunde pro Tag	☐
1/2 bis <1 Stunde pro Tag	☐
1 bis <2 Stunden pro Tag	☐
2 bis <3 Stunden pro Tag	☐
≥ 3 Stunden pro Tag	☐

7. Sich um eine ältere Person kümmern

gar keine	☐
< 1/2 Stunde pro Tag	☐
1/2 bis <1 Stunde pro Tag	☐
1 bis <2 Stunden pro Tag	☐
2 bis <3 Stunden pro Tag	☐
≥ 3 Stunden pro Tag	☐

8. Während Ihrer Freizeit am Computer zu sitzen

gar keine	☐
< 1/2 Stunde pro Tag	☐
1/2 bis <1 Stunde pro Tag	☐
1 bis <2 Stunden pro Tag	☐
2 bis <3 Stunden pro Tag	☐
≥ 3 Stunden pro Tag	☐

9. Fernzusehen oder ein Video anzuschauen

gar keine	☐
< 1/2 Stunde pro Tag	☐
1/2 bis <1 Stunde pro Tag	☐
1 bis <2 Stunden pro Tag	☐
2 bis <3 Stunden pro Tag	☐
≥ 3 Stunden pro Tag	☐

10. Sitzen und lesen, sprechen oder telefonieren, was nicht mit der Arbeit zu tun hat

gar keine	☐
< 1/2 Stunde pro Tag	☐
1/2 bis <1 Stunde pro Tag	☐
1 bis <2 Stunden pro Tag	☐
2 bis <3 Stunden pro Tag	☐
≥ 3 Stunden pro Tag	☐

11. Mit Tieren zu spielen

- gar keine ☐
- < 1/2 Stunde pro Tag ☐
- 1/2 bis <1 Stunde pro Tag ☐
- 1 bis <2 Stunden pro Tag ☐
- 2 bis <3 Stunden pro Tag ☐
- ≥ 3 Stunden pro Tag ☐

12. Leichtere Hausarbeiten zu verrichten – aufräumen, Betten machen, Wäsche waschen

- gar keine ☐
- < 1/2 Stunde pro Tag ☐
- 1/2 bis <1 Stunde pro Tag ☐
- 1 bis <2 Stunden pro Tag ☐
- 2 bis <3 Stunden pro Tag ☐
- ≥ 3 Stunden pro Tag ☐

13. Einkaufen zu gehen – Lebensmittel, Kleidung oder anderes

- gar keine ☐
- < 1/2 Stunde pro Tag ☐
- 1/2 bis <1 Stunde pro Tag ☐
- 1 bis <2 Stunden pro Tag ☐
- 2 bis <3 Stunden pro Tag ☐
- ≥ 3 Stunden pro Tag ☐

14. Intensivere Hausarbeiten zu verrichten – Böden kehren oder putzen, Fenster putzen

- gar keine ☐
- < 1/2 Stunde pro Woche ☐
- 1/2 bis <1 Stunde pro Woche ☐
- 1 bis <2 Stunden pro Woche ☐
- 2 bis <3 Stunden pro Woche ☐
- ≥ 3 Stunden pro Woche ☐

15. Moderat im Garten zu arbeiten – z.B. den Rasen auf einem Sitzrasenmäher zu mähen

- gar keine ☐
- < 1/2 Stunde pro Woche ☐
- 1/2 bis <1 Stunde pro Woche ☐
- 1 bis <2 Stunden pro Woche ☐
- 2 bis <3 Stunden pro Woche ☐
- ≥ 3 Stunden pro Woche ☐

16. Intensiver im Garten zu arbeiten – Rasen mähen oder harken

- gar keine ☐
- < 1/2 Stunde pro Woche ☐
- 1/2 bis <1 Stunde pro Woche ☐
- 1 bis <2 Stunden pro Woche ☐
- 2 bis <3 Stunden pro Woche ☐
- ≥ 3 Stunden pro Woche ☐

II. Ihre Fortbewegung im Alltag – Transportwege

Während dieses Trimesters, wie viel Zeit haben Sie normalerweise verbracht mit…

17. Langsam zu gehen, um z.B. zur Arbeit zu kommen oder den Bus zu erreichen – nicht zum Spaß oder als Fitnessübung

- gar keine ☐
- < 1/2 Stunde pro Tag ☐
- 1/2 bis <1 Stunde pro Tag ☐
- 1 bis <2 Stunden pro Tag ☐
- 2 bis <3 Stunden pro Tag ☐
- ≥ 3 Stunden pro Tag ☐

18. Schnell zu gehen, um z.B. zur Arbeit zu kommen oder den Bus zu erreichen – nicht zum Spaß oder als Fitnessübung

- gar keine ☐
- < 1/2 Stunde pro Tag ☐
- 1/2 bis <1 Stunde pro Tag ☐
- 1 bis <2 Stunden pro Tag ☐
- 2 bis <3 Stunden pro Tag ☐
- ≥ 3 Stunden pro Tag ☐

19. Mit dem Auto oder dem Bus zu fahren

- gar keine ☐
- < 1/2 Stunde pro Tag ☐
- 1/2 bis <1 Stunde pro Tag ☐
- 1 bis <2 Stunden pro Tag ☐
- 2 bis <3 Stunden pro Tag ☐
- ≥ 3 Stunden pro Tag ☐

III. Ihre Fortbewegung in der Freizeit – Spiel, Spaß und Sport

Wie viel Zeit verbringen Sie im Schnitt mit folgenden Tätigkeiten:

20. Langsam/gemütlich zu gehen – zum Spaß oder als Fitnessübung

- gar keine ☐
- < 1/2 Stunde pro Woche ☐
- 1/2 bis <1 Stunde pro Woche ☐
- 1 bis <2 Stunden pro Woche ☐
- 2 bis <3 Stunden pro Woche ☐
- ≥ 3 Stunden pro Woche ☐

21. Etwas schneller zu gehen – zum Spaß oder als Fitnessübung

- gar keine ☐
- < 1/2 Stunde pro Woche ☐
- 1/2 bis <1 Stunde pro Woche ☐
- 1 bis <2 Stunden pro Woche ☐
- 2 bis <3 Stunden pro Woche ☐
- ≥ 3 Stunden pro Woche ☐

22. Schnell bergauf gehen – zum Spaß oder als Fitnessübung

- gar keine ☐
- < 1/2 Stunde pro Woche ☐
- 1/2 bis <1 Stunde pro Woche ☐
- 1 bis <2 Stunden pro Woche ☐
- 2 bis <3 Stunden pro Woche ☐
- ≥ 3 Stunden pro Woche ☐

23. Joggen zu gehen

- gar keine ☐
- < 1/2 Stunde pro Woche ☐
- 1/2 bis <1 Stunde pro Woche ☐
- 1 bis <2 Stunden pro Woche ☐
- 2 bis <3 Stunden pro Woche ☐
- ≥ 3 Stunden pro Woche ☐

24. (Schwangerschafts-)Sportkurse zu besuchen

- gar keine ☐
- < 1/2 Stunde pro Woche ☐
- 1/2 bis <1 Stunde pro Woche ☐
- 1 bis <2 Stunden pro Woche ☐
- 2 bis <3 Stunden pro Woche ☐
- ≥ 3 Stunden pro Woche ☐

25. Schwimmen zu gehen

- gar keine ☐
- < 1/2 Stunde pro Woche ☐
- 1/2 bis <1 Stunde pro Woche ☐
- 1 bis <2 Stunden pro Woche ☐
- 2 bis <3 Stunden pro Woche ☐
- ≥ 3 Stunden pro Woche ☐

26. Tanzen zu gehen

- gar keine ☐
- < 1/2 Stunde pro Woche ☐
- 1/2 bis <1 Stunde pro Woche ☐
- 1 bis <2 Stunden pro Woche ☐
- 2 bis <3 Stunden pro Woche ☐
- ≥ 3 Stunden pro Woche ☐

27. Andere – bitte teilen Sie uns mit welche

____

- gar keine ☐
- < 1/2 Stunde pro Woche ☐
- 1/2 bis <1 Stunde pro Woche ☐
- 1 bis <2 Stunden pro Woche ☐
- 2 bis <3 Stunden pro Woche ☐
- ≥ 3 Stunden pro Woche ☐

28. Andere – bitte teilen Sie uns mit welche ______________________________

- gar keine ☐
- < 1/2 Stunde pro Woche ☐
- 1/2 bis <1 Stunde pro Woche ☐
- 1 bis <2 Stunden pro Woche ☐
- 2 bis <3 Stunden pro Woche ☐
- ≥ 3 Stunden pro Woche ☐

IV. Im Beruf

Wie häufig üben Sie nachfolgende Tätigkeit aus:

29. Zu sitzen – im Büro oder im Unterricht

- gar keine ☐
- < 1/2 Stunde pro Tag ☐
- 1/2 bis <1 Stunde pro Tag ☐
- 1 bis <2 Stunden pro Tag ☐
- 2 bis <3 Stunden pro Tag ☐
- ≥ 3 Stunden pro Tag ☐

30. Zu stehen oder langsam zu gehen, während Sie Dinge tragen, die schwerer als 4 kg sind

- gar keine ☐
- < 1/2 Stunde pro Tag ☐
- 1/2 bis <1 Stunde pro Tag ☐
- 1 bis <2 Stunden pro Tag ☐
- 2 bis <3 Stunden pro Tag ☐
- ≥ 3 Stunden pro Tag ☐

31. Zu stehen oder langsam zu gehen, ohne irgendetwas zu tragen

- gar keine ☐
- < 1/2 Stunde pro Tag ☐
- 1/2 bis <1 Stunde pro Tag ☐
- 1 bis <2 Stunden pro Tag ☐
- 2 bis <3 Stunden pro Tag ☐
- ≥ 3 Stunden pro Tag ☐

32. Schnell zu gehen, während Sie Dinge tragen, die schwerer als 4 kg sind

- gar keine ☐
- < 1/2 Stunde pro Tag ☐
- 1/2 bis <1 Stunde pro Tag ☐
- 1 bis <2 Stunden pro Tag ☐
- 2 bis <3 Stunden pro Tag ☐
- ≥ 3 Stunden pro Tag ☐

33. Schnell zu gehen, ohne irgendetwas zu tragen

- gar keine ☐
- < 1/2 Stunde pro Tag ☐
- 1/2 bis <1 Stunde pro Tag ☐
- 1 bis <2 Stunden pro Tag ☐
- 2 bis <3 Stunden pro Tag ☐
- ≥ 3 Stunden pro Tag ☐

VIELEN DANK FÜR IHRE TEILNAHME UND DIE MÜHEN!
